# Computational disease progression modeling can provide insights into cancer evolution

**DOI:** 10.18632/oncoscience.501

**Published:** 2020-05-01

**Authors:** Steve Goodison, Mark E. Sherman, Yijun Sun

**Affiliations:** ^1^Department of Health Sciences Research, Mayo Clinic, Jacksonville, FL, USA; ^2^Department of Microbiology and Immunology, The State University of New York, Buffalo, NY, USA; ^3^Department of Computer Science and Engineering, The State University of New York, Buffalo, NY, USA; ^4^Department of Biostatistics, The State University of New York, Buffalo, NY, USA

**Keywords:** machine learning, breast cancer, disease progression

The development of cancer from a single transformed cell to a biologically complex and potentially lethal disease proceeds through the expansion of divergent clonal lineages that establish distinct subpopulations [1]. This process can be viewed as a Darwinian, multistep evolutionary process at the cellular level driven by intrinsic cell characteristics, most notably the accumulation of genetic alterations, and selection pressures exerted by a co-evolving tumor microenvironment. An understanding of tumor evolution would provide valuable insights into tumor biology and establish a framework for the development of improved cancer taxonomies, prognostics and targeted therapeutics. Conceptual models of evolution have been inferred from the chronological ordering of mutations in single or related tumors, but established models derived from human tumor tissue data that describe cancer progression are lacking for most histotypes.

Time-series data are ideal for deriving models of dynamic progression, but this is impossible to collect in human cancer because of the need for timely treatment , which alters the natural history of the disease and exerts selection pressures that affect tumor evolution. Further, sampling itself may alter tumor biology, and small samples collected at a limited number of time points may provide incomplete or inaccurate representation of progression. In contrast, molecular profile data obtained from thousands of tumor tissues continues to accumulate, and computational methods to handle and interpret the high-dimensional and multi-platform data is keeping pace [2,3]. In order to leverage this data resource for potential cancer progression modeling, we devised a computational strategy [4] that derives pseudo time-series data from ‘static’ samples (single time-point tissue specimens). The design is based on the rationale that each sample provides a snapshot of the disease process, and analysis of a large number of samples can enable the development of a detailed model of disease progression that provides testable hypotheses. The computational approach, referred to as CancerMapp, was first applied to the analysis of transcriptome data from breast cancer consortium datasets [5,6].

Breast cancer is a heterogenous disease, composed of etiologically and clinically distinct subtypes: basal-like, luminal A, luminal B, and HER2+ [7]. Our modeling analysis revealed data structures that supported two distinct trajectories to aggressive phenotypes, either directly to the basal subtype, or through the luminal A and luminal B subtypes to the HER2+ subtype. The progression model was validated by the derivation of similar data structures in independent datasets, and by the mapping of additional genomic and clinical data on the gene expression model structure [4], revealing a clear trend of worsening survival function along the luminal progression trajectory. The unbiased computational approach supports the idea that breast cancer development follows limited, common progression paths, consistent with a two-component etiological model that depicts breast cancer arising from two main cell types of origin [8]. The interpretation from the bifurcating model is that the basal subtype is a distinct entity, and the luminal and HER2+ subtypes may be considered as different stages of the same disease process. Progression along the luminal trajectory likely includes elements of linear and branching evolution theory, but considering luminal A, Luminal B and HER2+ as a continuum of disease could have significant impacts on research design and subsequently, on clinical management. Moreover, this model would pose limitations for relating etiological exposures to specific molecular subtypes for cancers that have evolved.

As an example, we used the progression model as a framework to investigate the inter-relationships between paired primary and metastatic lesions [9]. We considered that primary and metastatic breast tumor tissue samples recovered from the same patient represent two time-points in a continuum of disease progression. The mapping of molecular profiles from these paired samples on to the progression model would enable the visualization of the evolutionary relationship between paired samples, and provide insight into disease progression. Analysis of gene expression data obtained from 246 matched primary and metastatic tumor samples [10] confirmed that basal lesions were distinct from luminal phenotypes, molecular phenotypes can shift within the same individual, and that the shift is unidirectional along a continuum of disease state towards malignancy, as postulated by the progression model [4]. As molecular analyses add to the growing wealth of descriptive molecular data related to cancer [11], modeling approaches such as CancerMapp will be needed to transform raw data into biological insights that have clinical relevance. Cancer progression models can overcome the constraints of mainstay prevalence-based approaches, providing an opportunity to detect genetic events directly based on cancer evolution theory, and thereby to reveal possible roles in the context of a dynamic disease process.

Interactive progression models and cancer roadmaps that can incorporate multi-platform data will also inform a new range range of research directions. For example, current prognostic tests in breast cancer are of value only in a restricted set of patients, but if we can visualize the entire, ordered progressive disease process, the identification of specific molecular characteristics associated with a broader spectrum of cancer phenotypes becomes feasible. With increasing use of genomic sequencing in clinical management, approaches that can place molecular changes into an evolutionary context would greatly facilitate the goal of personalized medicine and treatment success. Specifically, discriminating cancers that are in dynamic evolution to more aggressive subtypes from those that are more stable has potential implications for initial treatment, surveillance and prognosis. Future studies, using higher-resolution genomic methods, such as single-cell sequencing and tissue microdissection, guided by a working model, can provide data for the refinement of cancer progression roadmaps.
